# Next generation sequencing panel based on single molecule molecular inversion probes for detecting genetic variants in children with hypopituitarism

**DOI:** 10.1002/mgg3.395

**Published:** 2018-05-08

**Authors:** María I. Pérez Millán, Sebastian A. Vishnopolska, Alexandre Z. Daly, Juan P. Bustamante, Adriana Seilicovich, Ignacio Bergadá, Débora Braslavsky, Ana C. Keselman, Rosemary M. Lemons, Amanda H. Mortensen, Marcelo A. Marti, Sally A. Camper, Jacob O. Kitzman

**Affiliations:** ^1^ Institute of Biomedical Investigations (INBIOMED‐UBA‐CONICET) University of Buenos Aires Buenos Aires Argentina; ^2^ Department of Biological Chemistry (IQUIBICEN‐UBA‐CONICET) Faculty of Exact and Natural Sciences University of Buenos Aires Buenos Aires Argentina; ^3^ Department of Human Genetics University of Michigan Ann Arbor MI USA; ^4^ División de Endocrinología Hospital de Niños Ricardo Gutiérrez Centro de Investigaciones Endocrinológicas ‘Dr César Bergadá’ (CEDIE) CONICET – FEI Buenos Aires Argentina

**Keywords:** congenital hypopituitarism, GH1, growth hormone deficiency, single‐molecule molecular inversion probes

## Abstract

**Background:**

Congenital Hypopituitarism is caused by genetic and environmental factors. Over 30 genes have been implicated in isolated and/or combined pituitary hormone deficiency. The etiology remains unknown for up to 80% of the patients, but most cases have been analyzed by limited candidate gene screening. Mutations in the *PROP1* gene are the most common known cause, and the frequency of mutations in this gene varies greatly by ethnicity. We designed a custom array to assess the frequency of mutations in known hypopituitarism genes and new candidates, using single molecule molecular inversion probes sequencing (smMIPS).

**Methods:**

We used this panel for the first systematic screening for causes of hypopituitarism in children. Molecular inversion probes were designed to capture 693 coding exons of 30 known genes and 37 candidate genes. We captured genomic DNA from 51 pediatric patients with CPHD (*n* = 43) or isolated GH deficiency (IGHD) (*n* = 8) and their parents and conducted next generation sequencing.

**Results:**

We obtained deep coverage over targeted regions and demonstrated accurate variant detection by comparison to whole‐genome sequencing in a control individual. We found a dominant mutation *GH1*, p.R209H, in a three‐generation pedigree with IGHD.

**Conclusions:**

smMIPS is an efficient and inexpensive method to detect mutations in patients with hypopituitarism, drastically limiting the need for screening individual genes by Sanger sequencing.

## 1 INTRODUCTION

Pituitary dysfunction is an important human health problem that is caused primarily by congenital birth defects and pituitary adenomas. Hormone deficiencies can be isolated, and Isolated **g**rowth **h**ormone **d**eficiency (IGHD) is the most common, or involve two or more pituitary hormones: **c**ombined **p**ituitary **h**ormone **d**eficiency (CPHD). IGHD progresses to CPHD in 45% of patients (Blum et al., 2014; Otto et al., 2015). Only 16% of the cases of congenital CPHD can be explained by mutations in known genes (Cogan et al., 1998; Coya et al., 2007; Dateki et al., 2010; de Graaff et al., 2010; De Rienzo et al., 2015; Deladoey et al., 1999; Diaczok, Romero, Zunich, Marshall, & Radovick, 2008; Dusatkova et al., 2016; O. V. Fofanova et al., 1998; Halasz et al., 2006; Kandemir et al., 2012; Kim et al., 2003; Lebl et al., 2005; Lemos et al., 2006; McLennan et al., 2003; Mehta & Dattani, 2008; Navardauskaite et al., 2014; Pfaeffle et al., 2007; Rainbow et al., 2005; Reynaud et al., 2006; Takagi et al., 2012; Turton, Mehta, et al., 2005; Vieira, Boldarine, & Abucham, 2007), and for IGHD the rate is about 11% (Alatzoglou & Dattani, 2010; Wit et al., 2016). Molecular diagnosis is critical for predicting disease progression and risk of recurrence (Agarwal, Bhatia, Cook, & Thomas, 2000; Bottner et al., 2004; Fluck et al., 1998; Pernasetti et al., 2000). Some congenital cases of CPHD are associated with enlarged pituitary glands, and molecular diagnosis distinguishes these as benign and distinct from adenomas that appear similar on MRI, avoiding unnecessary intracranial surgery (Mendonca et al., 1999; Riepe et al., 2001). Unidentified hypopituitarism can result in infant death, and some types of hypopituitarism are progressive, leading to life‐threatening disorders secondary to hypoglycemia and adrenal insufficiency (30–33).

Congenital combined pituitary hormone deficiency (CPHD) arises from defects in pituitary development and is sometimes associated with extra pituitary abnormalities, such as cleft lip/palate, a short stiff neck, and hypoplastic optic nerves. For example, mutations in *HESX1* (OMIM reference number *601802) can cause septo‐optic dysplasia (SOD), CPHD, and IGHD (Dasen et al., 2001; Dattani et al., 1998; Gage et al., 1996), mutations in *OTX2* (*600037) can cause craniofacial abnormalities, including anophthalmia with or without IGHD or CPHD (Dateki et al., 2008; Diaczok et al., 2008; Matsuo, Kuratani, Kimura, Takeda, & Aizawa, 1995; Mortensen, MacDonald, Ghosh, & Camper, 2011; Mortensen, Schade, Lamonerie, & Camper, 2015; Nishida et al., 2003; Tajima et al., 2009), and mutations in *GLI2* (*165230) can cause holoprosencephaly, CPHD, or hypogonadism hypogonadotropic (HH) (Arnhold, Franca, Carvalho, Mendonca, & Jorge, 2015; Flemming et al., 2013; Franca et al., 2010). Mutations in *PROP1* (*601538) are the most common known cause of CPHD, accounting for 11% of total cases worldwide (Cogan et al., 1998; Deladoey et al., 1999; O. Fofanova et al., 2000; Rosenbloom et al., 1999; Wu et al., 1998). *Prop1* is the first pituitary‐specific gene in the transcriptional hierarchy of genes that cause CPHD, and it is essential for developing a normal stem cell pool and for stimulating stem cells to undergo an epithelial to mesenchymal transition‐like (EMT) process necessary for cell migration and differentiation (Perez Millan, Brinkmeier, Mortensen, & Camper, 2016). *Prop1* is necessary to activate expression of *Pou1f1* (*173110) (Sornson et al., 1996), and *POU1F1* is mutated in individuals with CPHD or IGHD (Radovick et al., 1992; Sobrier et al., 2016; Tatsumi et al., 1992; Turton, Reynaud, et al., 2005; Turton, Strom, Langham, Dattani, & Le Tissier, 2012) and no other clinical features. From these examples, it is clear that CPHD is part of a spectrum disorder that spans from severe abnormalities including holoprosencephaly (HPE) and septo‐optic dysplasia (SOD) to milder cases with hypogonadotropic hypogonadism or IGHD (Fang et al., 2016; Raivio et al., 2012). The most common genes implicated in IGHD are those encoding growth hormone (*GH1)* (*139250) and the growth hormone releasing hormone receptor (*GHRHR*) (*139191). Also, IGHD is sometimes caused by mutations in genes involved in early embryonic development, like *OTX2*,* HESX1*,* SOX2* (*184429)*,* and *SOX3* (*313430) (Alatzoglou et al., 2009; Kelberman et al., 2006).

The identification of genetic mutations is important for understanding the variability and progression of the disease, and as a foundation for the development of new treatments. Until recently, genetic testing was performed on a gene‐by‐gene basis, starting with the most likely candidate gene. With the incorporation of next generation sequencing technologies, it is now possible to test a large number of genes from several individuals in a single assay, reducing effort, costs and time. Here, we present a novel and cost‐effective approach to screen for coding mutations in known and suspected CPHD and IGHD risk genes, based upon single‐molecule molecular inversion probe sequencing (smMIPS) (Hiatt, Pritchard, Salipante, O'Roak, & Shendure, 2013). We established a panel of 67 genes associated with CPHD and IGHD in humans and mice, including new candidate genes found by analysis of *Prop1* mutant mice (Perez Millan et al., 2016). This panel targets 693 coding exons. We analyzed 51 pediatric patients from Argentina with CPHD or IGHD and their parents. We found a dominant mutation p.R209H in *GH1* in a three‐generation pedigree with isolated growth hormone deficiency type II. Using single molecule molecular inversion probes capture and deep sequencing is an efficient and inexpensive method to detect mutations in patients with hypopituitarism. Identifying these potential variants will make it feasible to predict clinical outcomes from genetic data, which is necessary for patient diagnosis and prognosis, and for assessing the risk of future affected individuals.

## 2 MATERIALS AND METHODS

### 2.1 Subjects

Whole blood was collected from 51 Argentinean patients belonging to 44 unrelated families diagnosed with IGHD or CPHD at the Hospital de Niños Ricardo Gutiérrez, Buenos Aires, Argentina. Samples were collected from unaffected parents and other relatives when feasible and warranted. All subjects were informed of the purpose of the study and their written consent was obtained. Parental consent was sought for patients under the age of 18. The study was approved by the Ethics Committee of Hospital de Niños Ricardo Gutiérrez, Buenos Aires, Argentina. The University of Michigan Institutional Review Board approved the use of anonymized DNA samples.

Patients were diagnosed with growth hormone deficiency (GHD) on the basis of abnormally low growth velocity and peak GH less than 4.8 μg/L after sequential arginine/clonidine pharmacological stimulation tests. Thyroid‐stimulating hormone (TSH) deficiency was diagnosed in individuals with free thyroxine <1.0 ng/dl with low or normal TSH levels TSH is ≤10 mU/L in patients under 2 months of age and ≤6.5 mU/L in older infants; ACTH deficiency was diagnosed based on low basal serum cortisol, <30.3 nmol/L in patients under 2 months of age, <58 nmol/L in patients between 2 and 6 months, and <165 nmol/L in older infants (Ballerini et al., 2010). Prolactin deficiency was considered in individuals with serum levels <2.5th centile for sex and age. Central diabetes insipidus was diagnosed when polyuria was associated with a urinary:plasma osmolarity ratio of <1.5 and the patient had a plasma osmolality >300 mosm/L. Gonadotropin deficiency was diagnosed in boys aged between 15 days and 6 months when serum luteinizing hormone (LH) and testosterone were <5th centile, <0.8 IU/L and <30 ng/dl, respectively. In girls from the age of 15 days to 2 years, gonadotropin deficiency was assumed when follicle‐stimulating hormone (FSH) levels were <1.0 IU/L (Braslavsky et al., 2015). In older patients, gonadotropin deficiency was defined as delayed or absent pubertal development with a low serum testosterone (< 3.47 nmol/L) associated with inappropriately low or normal LH and FSH levels. CPHD was defined as the presence of hormone deficiency affecting at least two anterior pituitary hormone‐producing cell types. Brain and Pituitary Magnetic Resonance Imaging (MRI) was performed in all patients.

### 2.2 Genomic DNA isolation

Genomic DNA was extracted from peripheral blood cells, using Puregene Blood kit (QIAGEN) according to the protocol provided by the manufacturer. The DNA was quantified using QuantiFluor^®^ dsDNA System (Promega) and the DNA concentration was normalized to 25 ng/μl for smMIPS assay. The ratio of absorbance at 260 nm and 280 nm was used to assess the purity of DNA. All DNA samples included in the panel have a 260/280 ratio between 1.8 and 2.1. To assess smMIPS accuracy, we included DNA from GM12878, a gold‐standard reference cell line, with publically available variant calls from deep whole genome sequencing (WGS) (Zook et al., 2014) (Coriell Institute for Medical Research, Camden, NJ).

### 2.3 Molecular inversion probes design, capture and sequencing

Sixty seven genes were included in the smMIPS panel to target 693 coding exons totaling 174.1 kb of coding sequence (File S1). This panel was designed targeting the coding exons (as defined by the UCSC Genome Browser, “Known Gene” table, hg19 build), padded by ≥ 25 bp in each direction to include exon‐intron boundaries. Design, preparation, and capture using smMIPS probes were performed as previously described (Yoon et al., 2015). Briefly, a library of smMIPS probes was designed for batch synthesis using custom python scripts. Probe sequences were synthesized on a single microarray as 150mers by CustomArray, Inc. smMIPS probes were PCR amplified from the resulting pool, using externally directed primers “mipPrep1F” and “mipPrep1R” (5′‐GGTAGCAAAGTGCAGATGTGCTCTTC‐3′, and 5′‐TGAACTCACACTGCTCTGAACTCTTC‐3′), digested overnight with EarI (NEB) to remove flanking amplification primers, purified with one volume SPRI beads supplemented with five volumes isopropanol, and eluted in Tris‐EDTA pH 8. For smMIPS captures, approximately 3 ng smMIPS probes were combined with 125 ng genomic DNA, in a reaction mixture including Ampligase DNA Ligase Buffer 1X (Epicentre), 0.4 μM dNTPs (NEB), 3.2U HemoKlentaq (NEB) and 1U Ampligase (Epicentre). After denaturation at 95°C for 10 min and incubation at 60°C for 20 hr, linear probes and the remaining genomic DNA were removed by exonuclease treatment with ExoI and ExoII (NEB). The captured material was amplified by PCR using barcoded primers. The resulting PCR products were pooled (120 samples) for one lane of paired‐end 100 bp sequencing on an Illumina HiSeq 2500 instrument at the University of Michigan Sequencing Core.

### 2.4 Data analysis pipeline

**Figure 1:**
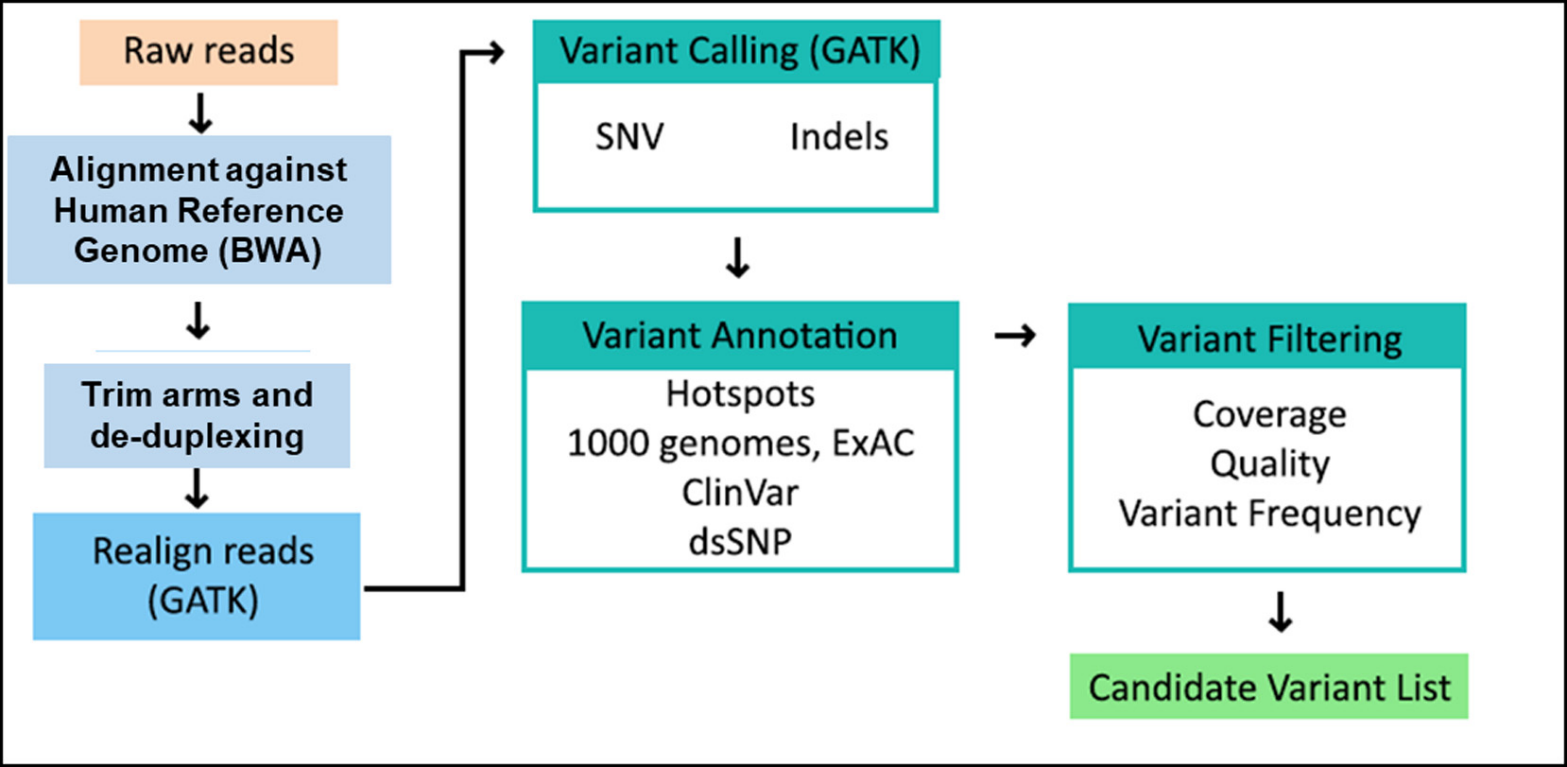
Bioinformatics pipeline and variant filtering strategy. Each step in the analysis of raw sequencing reads to development of a candidate variant list are indicated. Predicting functional effect of human missense mutations using PolyPhen‐2

We used a freely available, open source pipeline for smMIPS‐specific aspects of sequence alignment, downstream processing, and quality control (available at https://github.com/kitzmanlab/mimips). Briefly, this pipeline uses bwa‐mem (Li, 2013) to align reads to the human reference genome (build GRCh37), followed by custom python scripts to remove sequences derived from smMIPS probe oligonucleotides, and to remove reads with duplicate molecular tags (Figure  1). Variant calling was performed with Haplotype Caller from the Genome Analysis Toolkit (GATK) (McKenna et al., 2010) (DePristo et al., 2011) (Van der Auwera et al., 2013). The resulting VCF was further annotated with SnpEff/SnpSift (Cingolani et al., 2012), using the following main sources dbSNP, ExAC (Karczewski et al., 2017), ClinVar (Landrum et al., 2016), Polyphen (Adzhubei, Jordan, & Sunyaev, 2013), SIFT (Kumar, Henikoff, & Ng, 2009) and MutationTaster (Schwarz, Cooper, Schuelke, & Seelow, 2014). Variant prioritization was performed using our own developed variant analysis and prioritization software called B‐platform (http://www.bitgenia.com/b-platform/) following recent criteria from the *American College of Medical Genetics and Genomics* (ACMG) (Richards et al., 2015) to classify them. Depth of coverage was computed, using nonduplicate reads, and samples in which ≥80% of bases were covered at a threshold of ≥8 ×  coverage were considered passing.

For healthy controls, we use the ExAC database, which contains 123,136 exome sequences and 15,496 whole genome sequences from unrelated individuals without severe pediatric disease (gnomad@broadinstitute.org) (Lek et al., 2016), the online archive of Brazilian variants from 609 healthy individuals (http://abraom.ib.usp.br/), and Dr. Marti's private database of over 100 healthy Argentinean controls derived from our recent project (http://apps.bitgenia.com/100exomas).

### 2.5 Confirmation of *GH1* mutation by Sanger sequencing and CAP/CLIA clinical test

We amplified a 4 kb stretch of sequence including the *GH1* locus with the primers: 5′‐AAG TGA AAA GCA TCG AGA TGT GT‐3′ (*GH1* Forward) and 5′‐CAG CTA ACT TTT TTG CAT TTT TAG TAC AG‐3′ (GH1 Reverse). The reaction was run using Phusion‐based PCR (New England Biolabs, Ipswich, MA), with an annealing temperature of 67.0°C, and an extension time of 2 min. The resulting product was run on a 1% agarose gel, and a band of 4 kb was excised and purified, using a Qiagen Gel Extraction kit. Five nanograms of the extracted DNA were PCR amplified using primers that span exon 5 of *GH1*: 5′‐GGA CAC CTA GTC AGA CAA AAT GAT G‐3′ (*GH1* Exon 5 Forward) and 5′‐TCT CTA CAC CCT GAA GGG GAG‐3′ (*GH1* Exon 5 Reverse). The products were separated on a 1% agarose gel, and the 300 bp band was excised and purified in the same manner. Sixty ng of DNA at a concentration of 3 ng/μl were submitted to the University of Michigan sequencing core for Sanger Sequencing with the following primers:5′‐GAC ACC TAG TCA GAC AAA ATG ATG C‐3′ (*GH1* Sequencing Forward) and 5′‐AGG CTG GAA GAT GGC AGC‐3′ (*GH1* Sequencing Reverse). The chromatograms were analyzed to ensure amplification was specific to *GH1,* avoiding amplification of the paralogous genes *GH2* (*139240)*, CSH1* (*150200)*, CSH2* (*118820) and *CSHL1*(*603515)*. GH1* is distinguishable from *GH2* by adenine versus cytosine at the 589^th^ position of the mRNA. *GH1* is distinguishable from *CSH1* and *CSHL1*, by cytosine versus guanine at the 658^th^ position of the mRNA. Finally, *GH1* is distinguished from *CSH2* by polymorphic loci starting at position 715. Genomic DNA sequence of *GH1* was based on the GenBank reference sequences NG_011676.1.

## 3 RESULTS

### 3.1 Patient characteristics

The clinical features of 51 patients with CPHD or IGHD are summarized in Table 1. The median age of the patients was 9 years (range 1–29 years), and they represent 44 independent pedigrees with no consanguinity. A majority of these patients were diagnosed with CPHD (84%) and were sporadic cases. There were three familial cases including a three‐generation Caucasian pedigree with IGHD. Twenty‐five percent of the cases were native Argentineans or Amerindian descent.

**Table 1:** Characteristics of the study subjects

Total patients	51
Age	
Median age (range)	9 (1‐29)
Mean age	10.8
Gender	
Male	28 (55%)
Female	23 (45%)
Ethnicity	
Native	13 (25%)
Caucasian	38 (75%)
Diagnosis	
IGHD	8 (16%)
CPHD	43 (84%)
Cases	
Familial	3 (10 affected)
Sporadic	41
Pituitary hormone deficiency	
GH deficiency	51 (100%)
ACTH deficiency	30 (59%)
TSH deficiency	31 (61%)
Gonadotropin deficiency	13 (25%)
PRL deficiency	9 (18%)
ADH deficiency	2 (4%)
MRI: Pituitary stalk	
Absent	13
Thin	8
Interrupted	3
Normal	9
MRI: Anterior pituitary	
Absent	3
Hypoplasia	30
Normal	8
MRI: Posterior pituitary	
Absent	11
Ectopic	16
Normal	9

### 3.2 Single‐molecule molecular inversion probe (smMIP) sequencing panel

We developed a refined version of the single‐molecule molecular inversion probe (smMIPS) capture assay (Hiatt et al., 2013). The panel was designed to cover all coding exons and intron‐exon boundaries of 67 selected genes associated with CPHD, IGHD, SOD, and HPE in humans and/or mice (File S1). This panel targets 693 coding exons totaling 174.1 kb of coding sequence. Here, smMIPS capture, library preparation, and sequencing was performed for all 120 samples, using specific barcodes for each sample (Figure 1).

To assess smMIPS accuracy, we included DNA from GM12878, a gold‐standard reference cell line, with publically available variant calls from deep whole‐genome sequencing (WGS) (Zook et al., 2014). For this individual, we obtained 2.1 million read pairs, resulting in median coverage of 154X, and 97.6% of targeted bases reaching ≥8×  read depth coverage, and 95.1% of bases reaching ≥40×. Within regions with sufficient coverage, variant calling was highly accurate, with 99.54% SNP/indel variant sensitivity, with an overall genotype concordance of >99.6% (positions with ≥ 8 reads). After instituting genomic DNA quality control for concentration and absorbance ratio (260/280), and, as needed, re‐purification, 97% of samples sequenced successfully (defined as 98% of targeted bases at covered by ≥8 reads which is sufficient for sensitivity and specificity in the cell line). On average, 98% of regions of interest were covered >100×. Nine exons were not covered or had an average coverage lower than 10 (Figure S1).

### 3.3 Identification of *GH1* mutation

**Figure 2:**
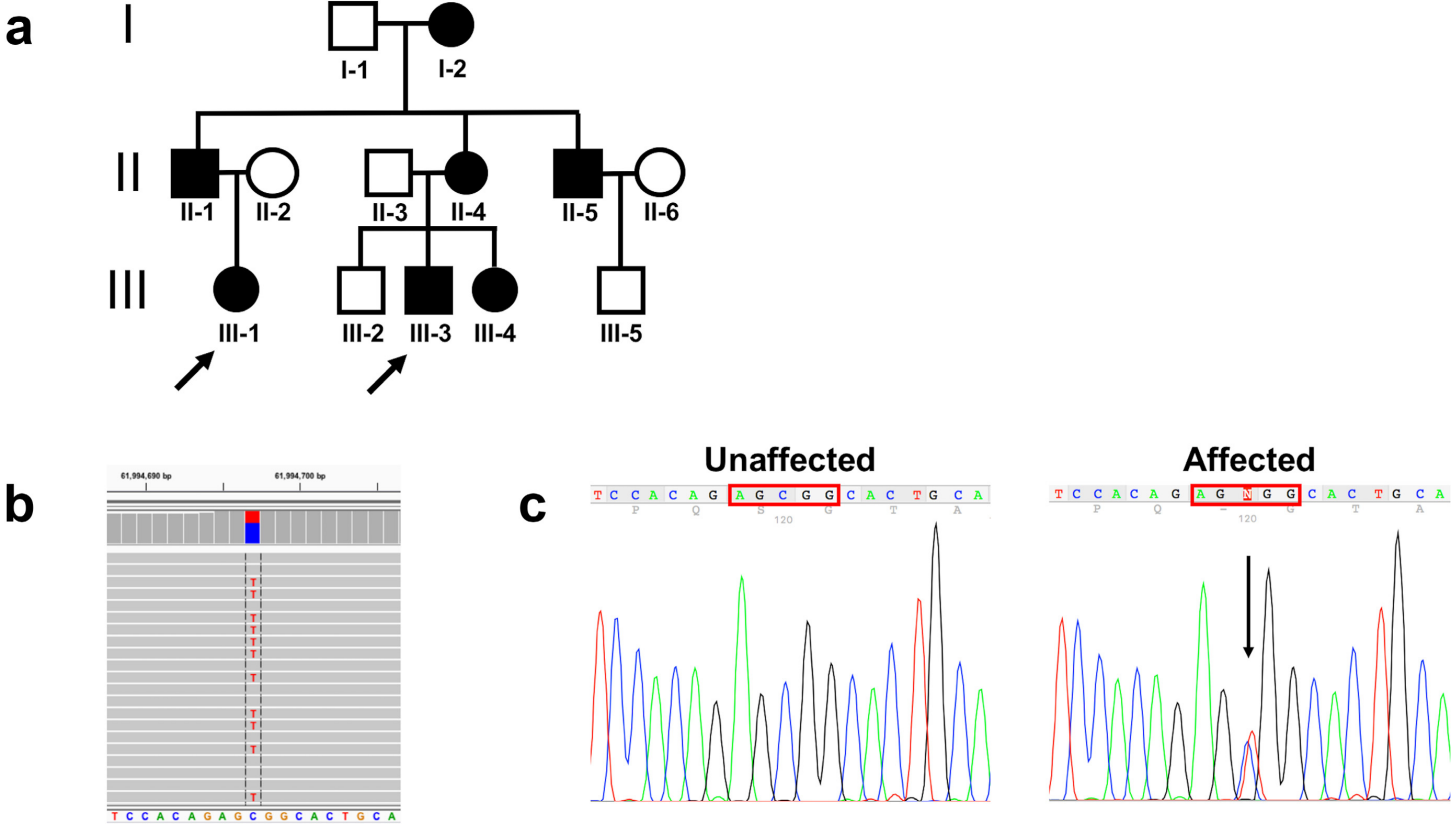
Pedigree and sequencing chromatograms. (a) Pedigree indicates autosomal dominant inheritance. The index patients are indicated with arrows. (b) Genome viewer detection of heterozygous G1664A (C>T) on reverse complement. (c) A sequence chromatogram showing the *GH1* (c.626G>A; p.R209H) mutation. In the chromatogram, the pathogenic variant is indicated with an arrow

We found a *GH1* mutation, in a three‐generation pedigree with autosomal dominant growth insufficiency, using smMIPS (Figure 2). MRI showed mild anterior pituitary hypoplasia in two patients and a thin pituitary stalk in one of them. We also found the same mutation in an apparently unrelated female patient with IGHD and in her apparently unaffected father who is deceased and no additional details are available. We confirmed proper segregation of the variant in the three generation pedigree with Sanger sequencing (Table 2). While this was in progress, a new baby was born in the family (III‐4). We arranged for a CAP/CLIA clinical test to be conducted so that results could be returned to the physicians. This test revealed that the baby was affected, and GH treatment began immediately. This example provides proof of the principle that the smMIPS can detect clinical relevant mutations in known genes. Patients III‐1 and III‐3 responded to GH treatment commencing at 9 years of age (mg kg^−1^w^−1^) and 4 years of age (mg kg^−1^ w^−1^) respectively.

**Table 2:** Clinical data of the families evaluated

Family	Gender	Diagnosis	Height (SDS)	Max peak GH (ng/ml)	IGF1 (SDS)	Mutation p.Arg183His
I‐2	F	IGHD	−2.5	NA	−8.3	Yes
II‐1	M	IGHD	−2.9	2.3	ND	Yes
II‐2	F	Normal	0.6	NA	NA	No
II‐3	M	Normal	1.5	NA	NA	No
II‐4	F	IGHD	−3.1	NA	−4.8	Yes
II‐5	M	IGHD	−3.2	NA	−3.7	Yes
III‐1	F	IGHD	−3.5	3.6	−6	Yes
III‐3	M	IGHD	−2.9	3.01	ND	Yes
III‐5	M	Normal	0.35	NA	2.3	No

NA, not available; ND, not detectable.

This mutation, C>T c.626G>A p.R209H based on ENST00000323322, has been described previously as p.R183H in several pedigrees and shown to interfere with the secretion of GH (Deladoey, Stocker, & Mullis, 2001; Gertner, Wajnrajch, & Leibel, 1998; Marino et al., 2003; Miyata et al., 1997). The numbering in the previous publication was based on assigning the first amino acid of the GH protein following cleavage of the signal peptide.

The frequency of the *PROP1* mutation varies widely by population group, and the rate was previously unknown for Argentina. We found no cases of *PROP1* mutations in this first cohort analyzed by smMIP selection and high throughput sequencing.

## 4 DISCUSSION

We developed a targeted next‐generation sequencing panel using single molecule molecular inversion probes (smMIPS) to identify mutations in pituitary hormone deficiency patients. Here, smMIPS is a rapid, scalable and economical method for sequencing candidate loci for mutation discovery and smMIPS enables multiplexed sequencing of targets ranging from small gene panels (Hor et al., 2015) to whole exomes (Turner, Lee, Ng, Nickerson, & Shendure, 2009) across very large cohorts for which whole‐genome or whole‐exome sequencing would be cost‐prohibitive. However, smMIPS have been previously used to screen for de novo mutations in autism risk genes, allowing interrogation of much larger cohorts than presently feasible with whole‐genome or exome sequencing (Neale et al., 2012; Stessman et al., 2017; Wang et al., 2016). And, smMIPS sequencing has also recently been applied clinically to test for mutations in the tumor suppressor genes *BRCA1* and *BRCA2* (Neveling et al., 2017) and has demonstrated superior accuracy and turnaround time relative to previous laboratory‐developed testing. We are not aware of systematic screening for pathogenic variants that cause CPHD or IGHD with panels of known genes in Argentina or any other population group.

Isolated growth hormone deficiency is most frequently caused by mutations in the *GH1* gene, especially gene deletions and conversion events stimulated by the array of GH related genes (Mullis, 2011). Pathogenic mutations in the growth hormone releasing hormone receptor, *GHRHR* (Salvatori et al., 1999, 2001; Wajnrajch, Gertner, Harbison, Chua, & Leibel, 1996) and *GHSR* have also been reported to cause IGHD (Inoue et al., 2011; Pantel et al., 2009; Pugliese‐Pires et al., 2011). IGHD1A and IGHD1B exhibit autosomal recessive mutations in *GH1*, while IGHD2 is characterized by autosomal dominant mutations in *GH1* (Phillips & Cogan, 1994). Individuals with IGHD2 present with variable height deficits and variable pituitary size, and other hormone deficits may emerge. The majority of these dominant cases are caused by mutations in the intron 3 splice donor site, which cause skipping of exon 3 and generation of a 17.5 kDa GH instead of the bioactive 22 or 20 kDa forms (Mullis et al., 2005). The 17.5 kDa form of GH has a dominant negative effect on GH secretion and causes cell death, explaining the progressive hormone deficiency (McGuinness et al., 2003; Ryther et al., 2003; Shariat, Holladay, Cleary, Phillips, & Patton, 2008). Mutations in exonic splice enhancers also cause increased production of the 17.5 kDa GH. There are a few missense mutations that cause IGHD2, and some of them are likely pathogenic because they affect splicing (Babu et al., 2014).

Our screening uncovered a recurrent *GH1* missense mutation, p.R209H, in a family with IGHD2 and in an unrelated sporadic case of IGHD. This recurrent mutation has been reported in ethnically diverse families with IGHD2 and some sporadic IGHD cases (previously referred to as p.R183H). It was reported in a three‐generation Turkish pedigree of Kurd ancestry (Deladoey et al., 2001), in two, large, unrelated families of Christian‐Arab and Ashkenazi Jewish descent (Hess et al., 2007), and in two unrelated IGHD patients from Argentina (Marino et al., 2003). Individuals with this variant exhibit a variable phenotype, with carriers of the same family exhibiting height (SDS) ranging from −4.5 to −1.0 (Hess et al., 2007). While all the variant carriers in the familial case reported here had severe short stature, the sporadic case had an apparently unaffected father, consistent with reports of variable expressivity of this allele. No additional pituitary hormone deficiency was found in our patients, and no progression has been reported for other patients with the same variant. All patients responded well to growth hormone replacement therapy.

The exact mechanism whereby the p.R209H GH impairs growth is not clear. However, elegant transfection studies demonstrated that the variant GH protein can be secreted effectively in response to cAMP stimulation, but if co‐expressed with the normal protein, secretion is greatly reduced (Deladoey et al., 2001). This suggests that the missense mutation interferes with the aggregation of GH proteins that is necessary to form secretory granules.

The frequency of *PROP1* mutations varies greatly based on ethnicity, with high levels reported in Lithuanian (65%) and Russian (46%) cohorts and less than 1% in patients from the United Kingdom, Germany, Japan and Korea (De Rienzo et al., 2015; Dusatkova et al., 2016; Navardauskaite et al., 2014). The Argentinean population is a mixture of European (67%), Native American (28%), West African (3.6%) and East Asian (1.4%) ancestry, and the European component is predominantly from Spain and Italy (Homburger et al., 2015). The rate of *PROP1* mutations in Argentina was 0/44, which compares well with the low rates of *PROP1* mutations in Spain (Coya et al., 2007) (0/36) and Italy (De Rienzo et al., 2015) (3/126, 2.4%). Slightly higher rates were reported for Portugal (9/36, 25%) (Lemos et al., 2006)) and Brazil (Vieira et al., 2007) (5/29, 17%).

In summary, we developed a gene panel based on single molecule molecular inversion probe sequencing and captured the coding exons of 67 candidate genes in 51 patients with hypopituitarism. We found a mutation in the *GH1* gene that is responsible for familial isolated growth hormone deficiency type II. Identifying these potential variants will make it feasible to predict clinical outcomes from genetic data, which is necessary for patient diagnosis and prognosis, and for assessing the risk of future affected individuals. We believe that the approach described here is cost and time efficient, and should be apply first in molecular diagnosis, follow by CNV assays and whole genome sequencing to provide much needed diagnoses for patients and their families.

## CONFLICT OF INTEREST

The authors have nothing to disclose. SAC, AZD, AHM, SV, JB, MIPM, AS, MM, IB, DB, AK, and JOK.
