# Supplementary figures and images for: Next generation sequencing panel based on single molecule molecular inversion probes for detecting genetic variants in children with hypopituitarism
Source: Mol Genet Genomic Med. 2018 May 8;6(4):514–25. doi: 10.1002/mgg3.395 (PMC6081231; doi:10.1002/mgg3.395)

Figure S1 I

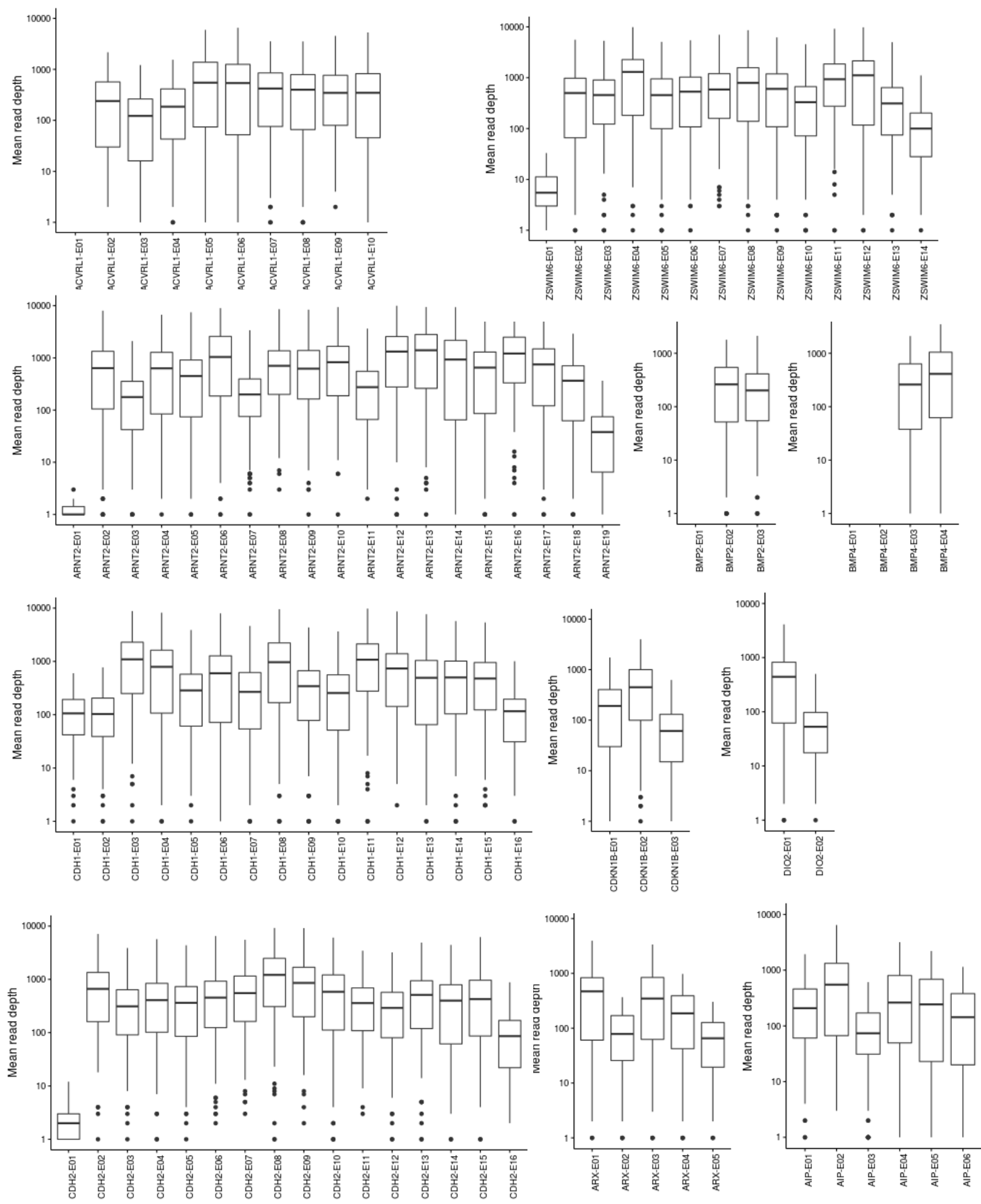

Supplement: Supplementary file 1 [file MGG3-6-514-s001.pdf]

Figure S1 II

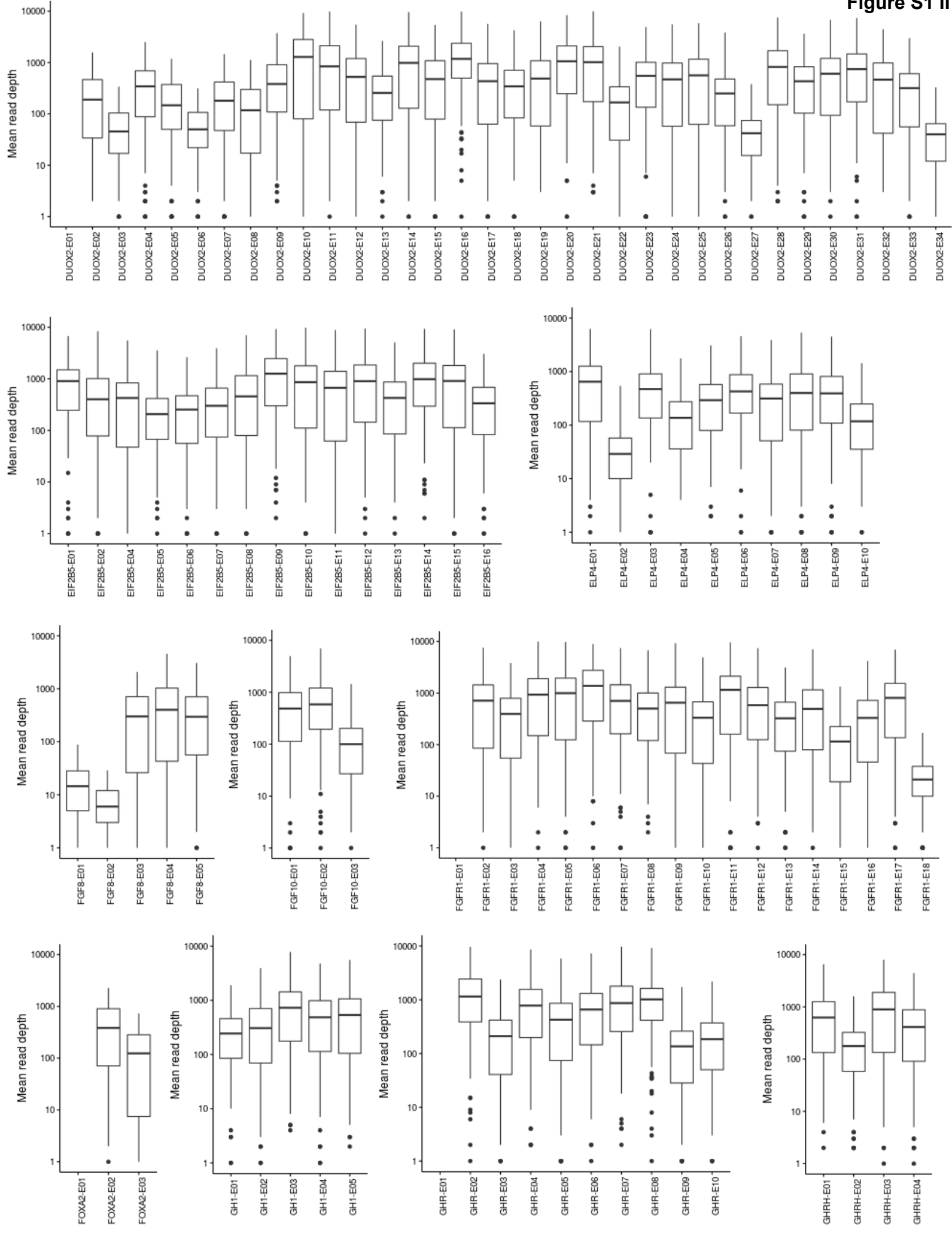

Supplement: Supplementary file 2 [file MGG3-6-514-s002.pdf]

Figure S1 III

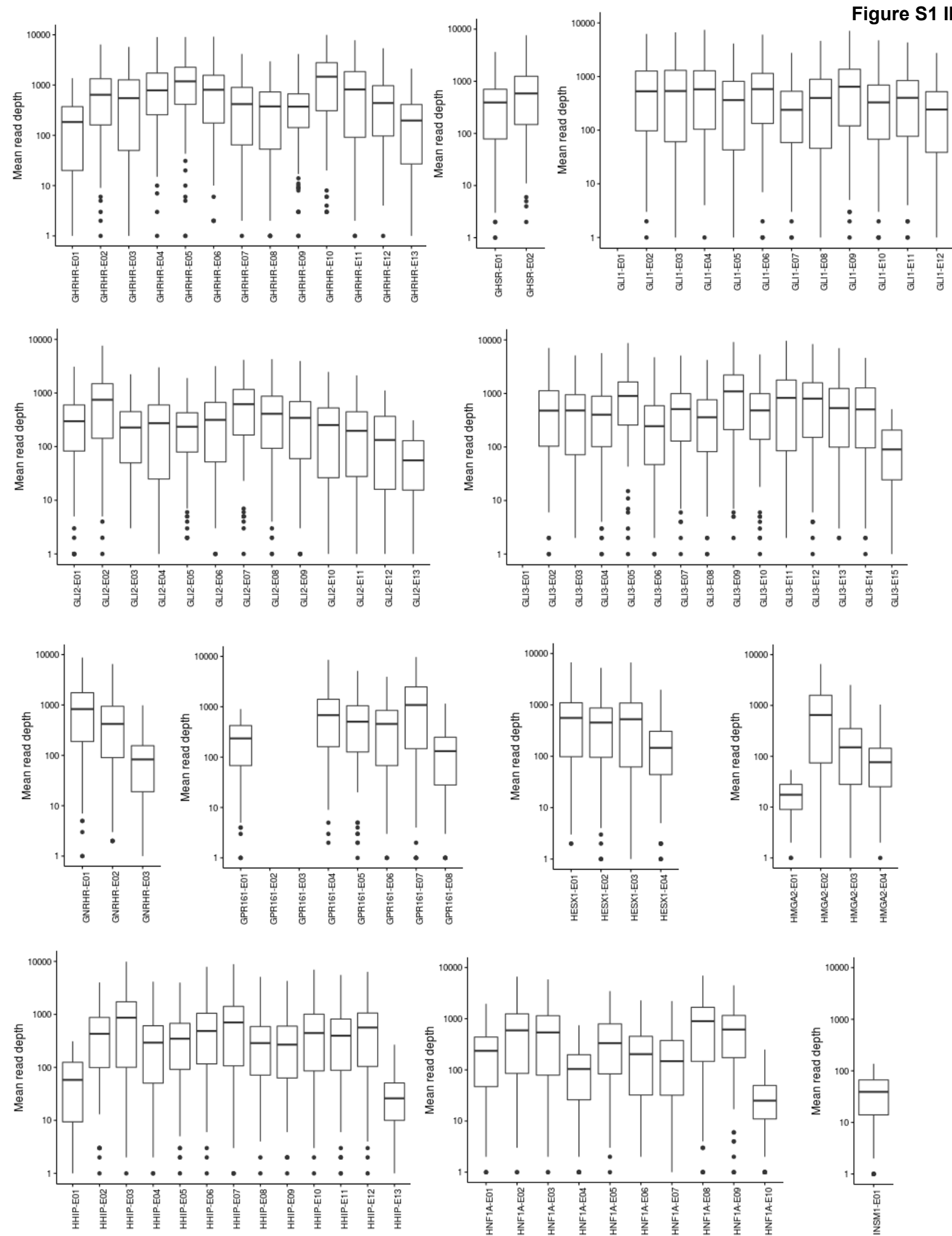

Supplement: Supplementary file 3 [file MGG3-6-514-s003.pdf]

Figure S1 IV

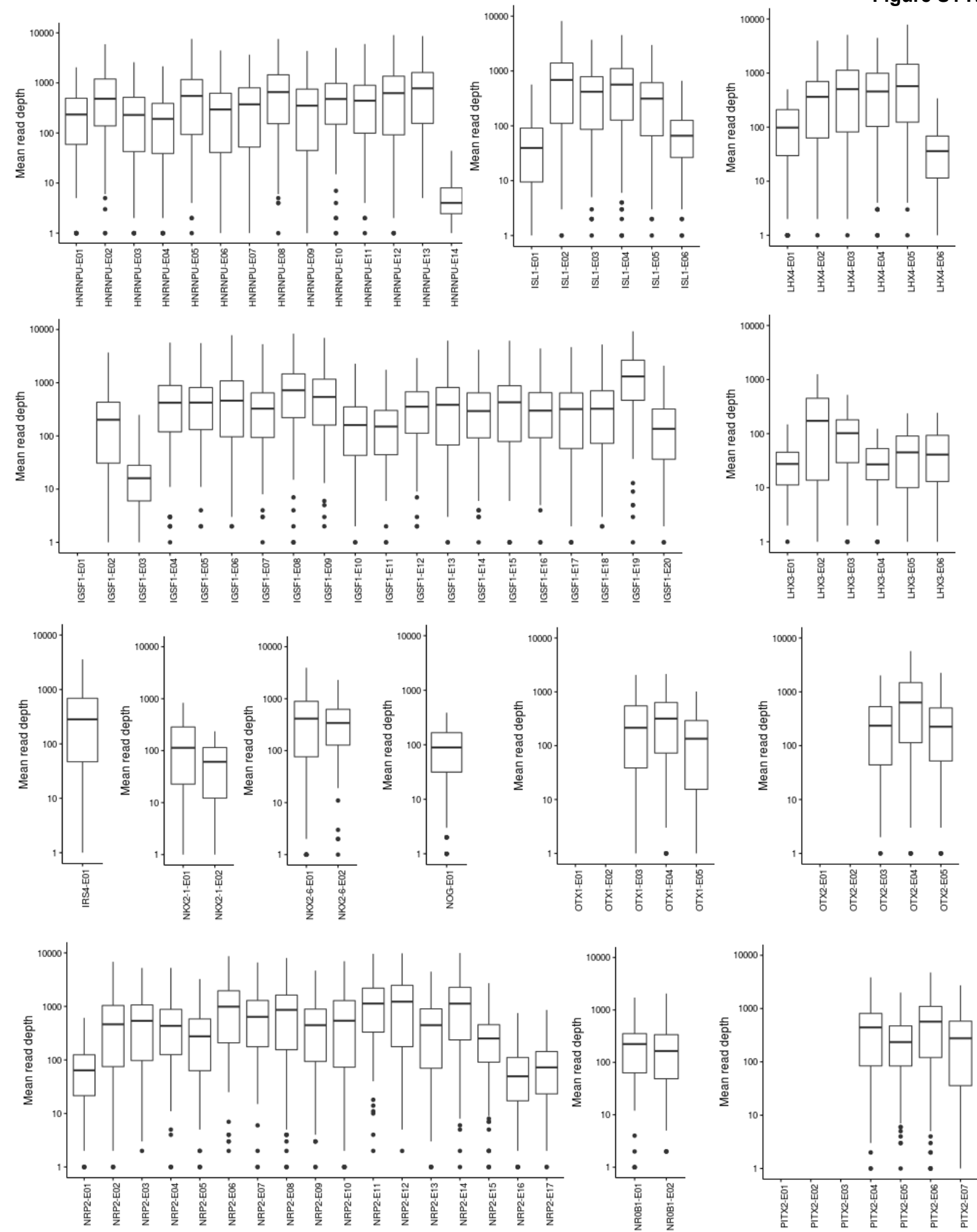

Supplement: Supplementary file 4 [file MGG3-6-514-s004.pdf]

Figure S1 V

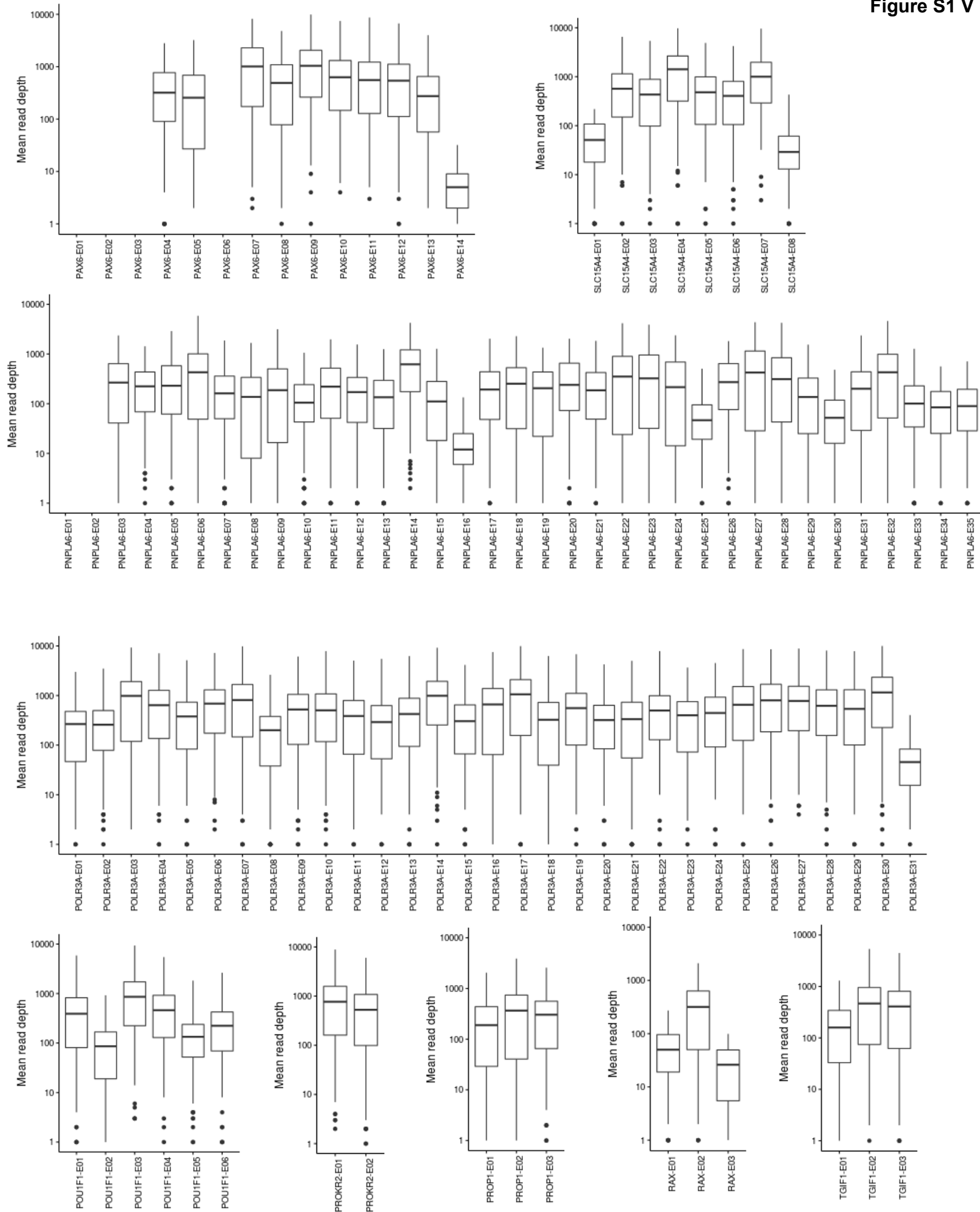

Supplement: Supplementary file 5 [file MGG3-6-514-s005.pdf]

Figure S1 VI

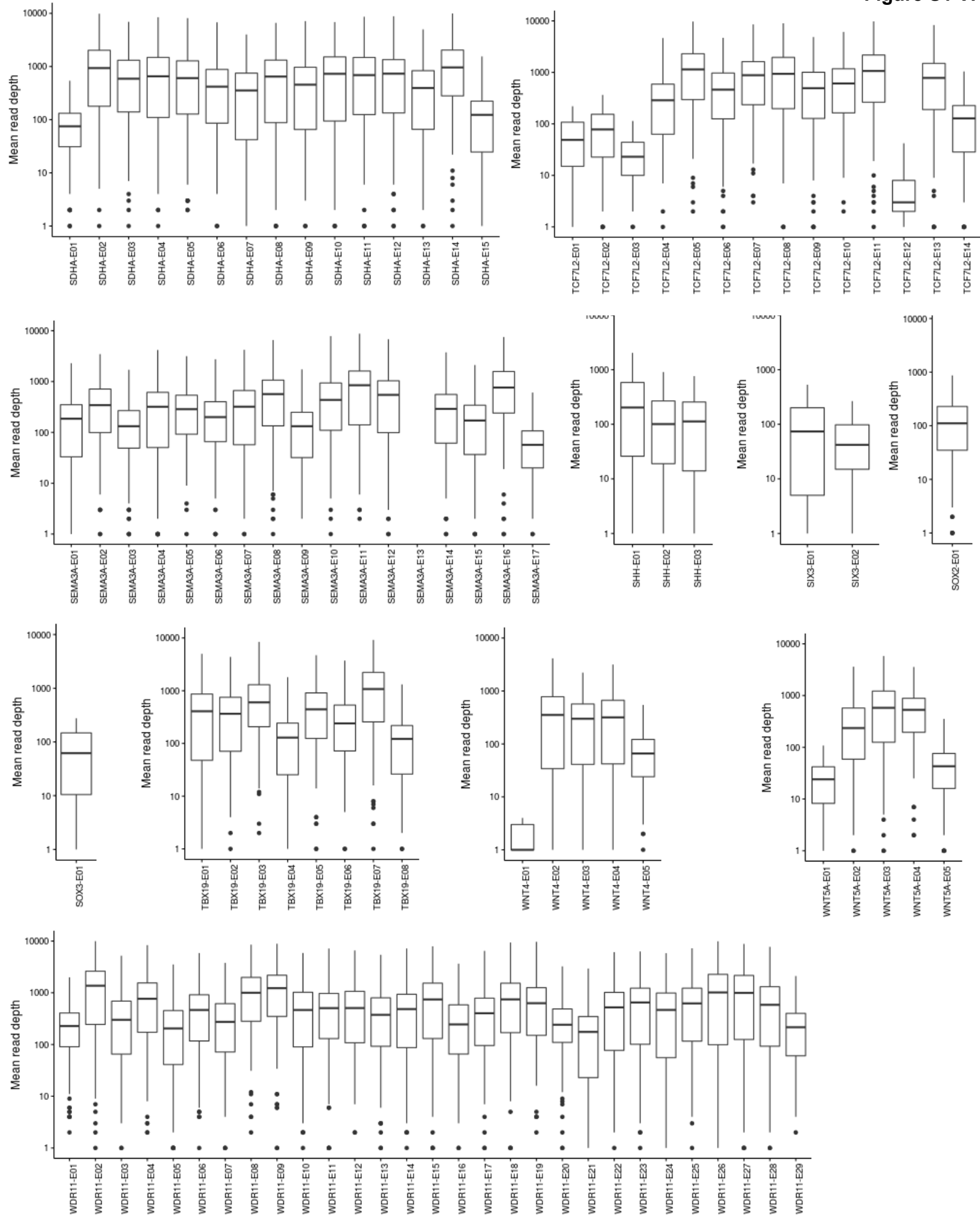

Supplement: Supplementary file 6 [file MGG3-6-514-s006.pdf]
